# Supplementary material for: Quantifying the phenotypic information in mRNA abundance
Source: Mol Syst Biol. 2022 Aug 15;18(8):e11001. doi: 10.15252/msb.202211001 (PMC9376724; doi:10.15252/msb.202211001)
Supplement: Supplementary file 1 — Appendix S1 [file MSB-18-e11001-s002.pdf]

Appendix

Table of Content:

1..... Appendix Figure S1

2..... Appendix Figure S2

3..... Appendix Figure S3

4..... Appendix Figure S4

MINE estimates mutual information by calculating the KL Divergence between the marginal distributions and the joint distribution. This KL divergence represents the distance between these distributions, which is nonnegative such that 0 represents complete independence. MINE uses the Donsker-Varadahn representation of the KL Divergence to evaluate a function that maps samples of the data to the set of all real numbers. Gradient ascent finds the parameterization of this function which maximizes the mutual information for a tight lower bound.

To validate MINE's estimates before applying it to the real data, we evaluated MINE on multivariate gaussian distributions with an analytically calculable mutual information between toy genes and toy Ca^{2+} (I(TG;TC)). The "toy" data was created using a standard additive white gaussian noise (AWGN) model with tunable dependency, entropy, and dimensionality (SF 1). A multivariate gaussian distribution (5128,16) was defined based on a specified covariance structure (SF 1B). The covariance of the distribution is somewhat arbitrary; it is only required that the matrix is invertible and tunable over a range of dependencies. The covariance structure is shown as 4 symmetrical quadrants in the matrix: the off diagonal quadrants are -α and the on-diagonal quadrants are α (SF 1A). Toy signals were created by applying a tunable amount of noise to the toy genes (SF 1C).

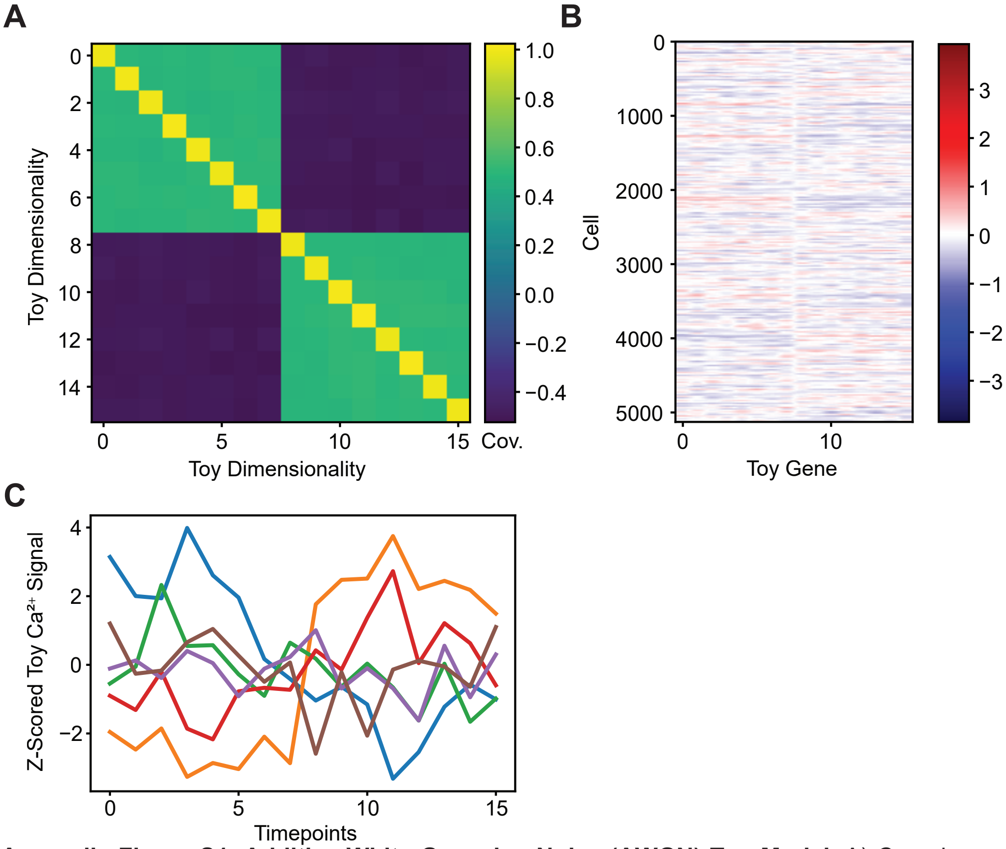

Appendix Figure S1. Additive White Gaussian Noise (AWGN) Toy Model. A) Covariance matrix for toy data. Quadrant values are determined by a hyperparameter α, where off-diagonal quadrants are set to -α and on-diagonal quadrants are set to α, with variance set to 1. B) Toy genes are determined by the covariance matrix. C) Toy signals are calculated by adding a tunable amount of noise to the toy gene matrix: $TC = TG + \mathcal{N}(\mu, n\sigma_G^2)$.

Evaluating MINE on the toy data, we found that the model occasionally produces a non-converging, biased mutual information estimate that increases linearly with training (SF 2A). To quantify and remove this bias, we fit MINE's output to a statistical model: $I_{obs}(t) = I_{true}(1-a) * e^{-b*t} + ct$, where I_{true}, a, b, and c are the fitting parameters and t is the number of iterations. Finding the optimal I_{true} factors out the linear bias c and produces a converging and accurate estimate of the analytically determined I(TG;TC) (SF 2B). The mean over the three replicates yields a final slope of 5.8e-6, showing reliable, asymptotic convergence to the true mutual information value. Applying this solution allowed rigorous definition of a convergence criterion that adaptively determines when training can conclude (SF 2C). This criterion uses moving averages to determine convergence, and expectedly results in higher residuals for fits with fewer iterations (SF 2D). Stricter convergence thresholds can fail to converge because of noise in the output; 1.4 was chosen as the final threshold due to its high yield, fast convergence, and low mean residual (SF 2E). In addition to the low residuals, we also found a pearson correlation of 0.97 across a range of I(TG;TC) (SF 2F). These results support the use of our bias-corrected MINE with the chosen hyperparameters.

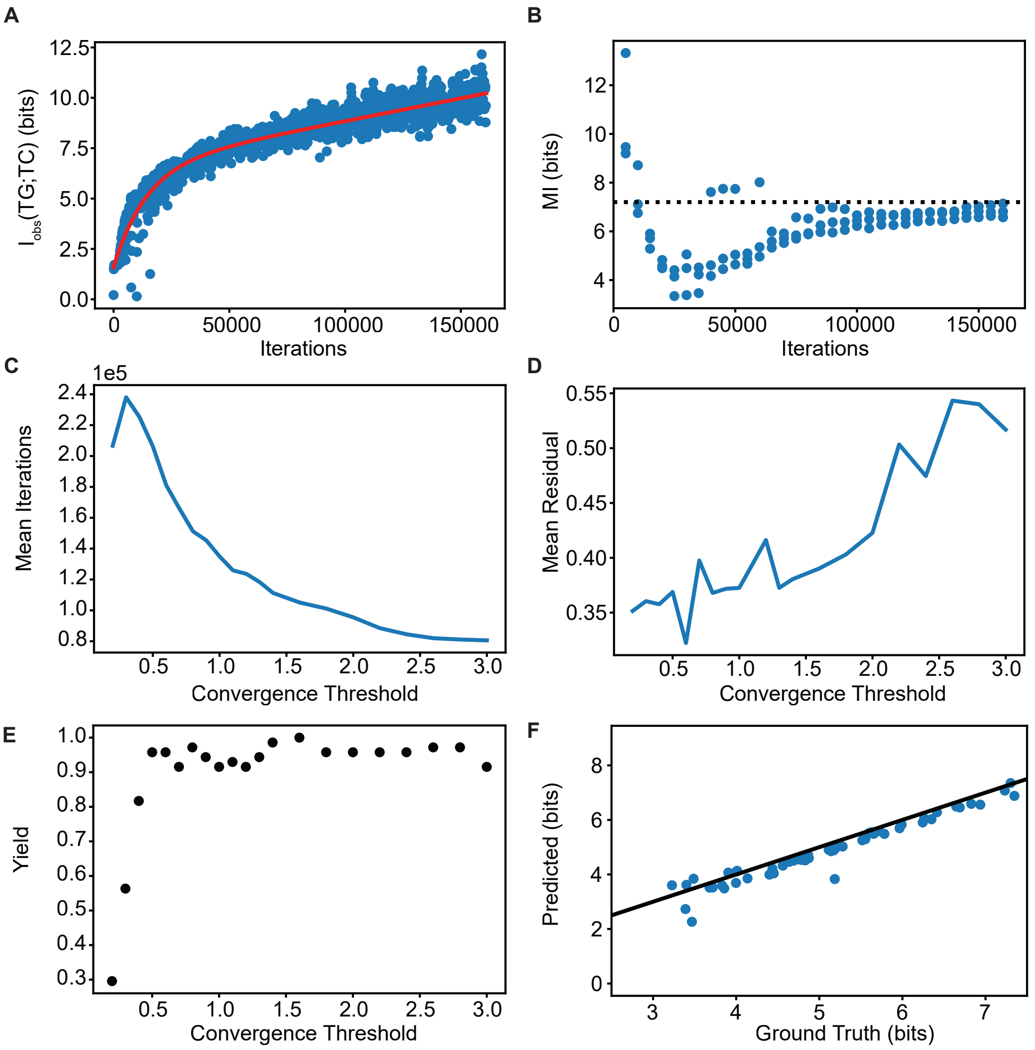

Appendix Figure S2. Fitting Model on Toy Data. A) Blue points are the raw output from MINE. The model occasionally failed to converge at the analytical I(TG;TC) value, as shown. Bias correction fit using BFGS optimization is shown in red. B) Blue points show the bias correction estimate of the true I(TG;TC) during training from 3 replicates. The dotted black line shows the true, analytical I(TG;TC) value. C) An adaptive convergence criterion based on the difference between moving averages was used to decrease training time. Higher convergence thresholds, i.e. larger differences between moving averages, result in fewer iterations. D) Faster convergence expectedly results in poorer fits with higher residuals on average. E) If the convergence threshold is too stringent, the optimization algorithm may be unable to find a suitable fit for bias correction. The yield was calculated over many toy datasets with a range of parameter values. F) Toy models with a large range of analytical mutual information values were fit using bias-corrected MINE, pearson *r* = 0.97.

We then applied bias-corrected MINE to the full data. We found that the bias correction works well on the real data and eventually converges on a single value (SF 3A). To verify that the results are not limited by sample size, we performed a jackknife correction (SF 3B). The jackknife extrapolation to infinite sample size yielded a result well within the error bars of the estimate at the full sample size. This result indicates that sample size is not limiting.

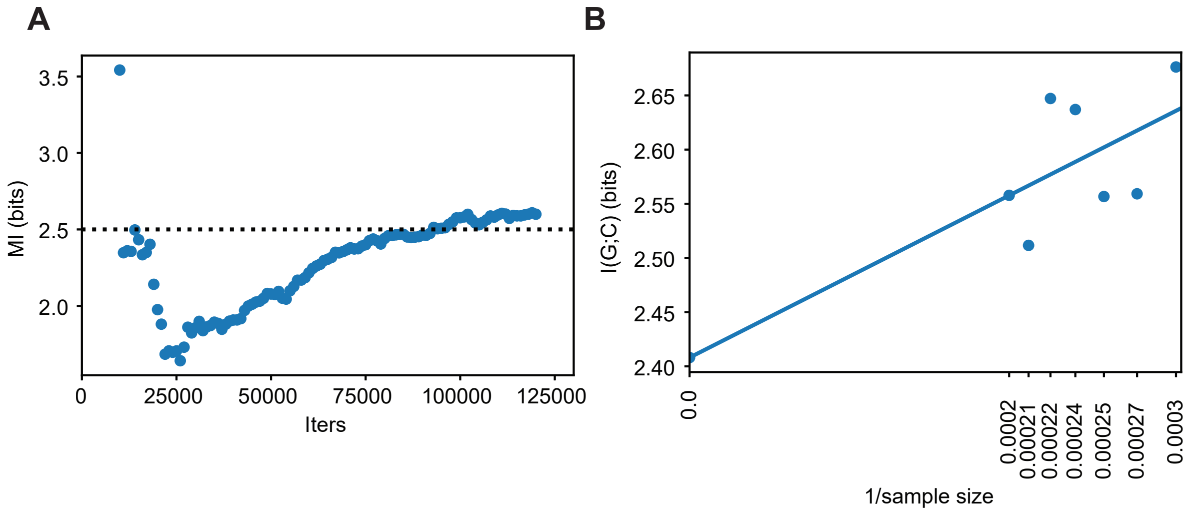

Appendix Figure S3. Bias-Corrected MINE on Real Data. A) Full data was fit using bias-correction and the estimate of I(G;Ca^{2+}) is shown in blue. The dotted black line is the mean of several samples. B) Jackknife of the data with 7 sample sizes ranging from 3369 to 5128 (all cells). The intercept shown is the extrapolation at infinite sample size, which is well within the estimate of 2.5 ± 0.4 bits.

To estimate the upper bound on the MI, we used FFT spectral entropy. This calculation begins by creating an FFT periodogram (Fig 1D). The Shannon entropy of the resulting distribution of power spectral densities represents the spectral entropy. To verify that spectral entropy produces an invariant measurement of entropy unlike other differential entropy metrics, we first applied various transformations to the signals. These transformations did not change the final entropy, whereas smoothing by convolution did degrade the entropy, as expected (SF 4A). Furthermore, we applied linear interpolations to change the dimensionality and found that most of the entropy comes from low frequencies which are well preserved even with dramatic reduction in timepoints (SF 4B). Increasing the dimensionality by interpolation did not change the entropy estimate. Therefore, we conclude that spectral entropy is a robust, invariant measure of signal information.

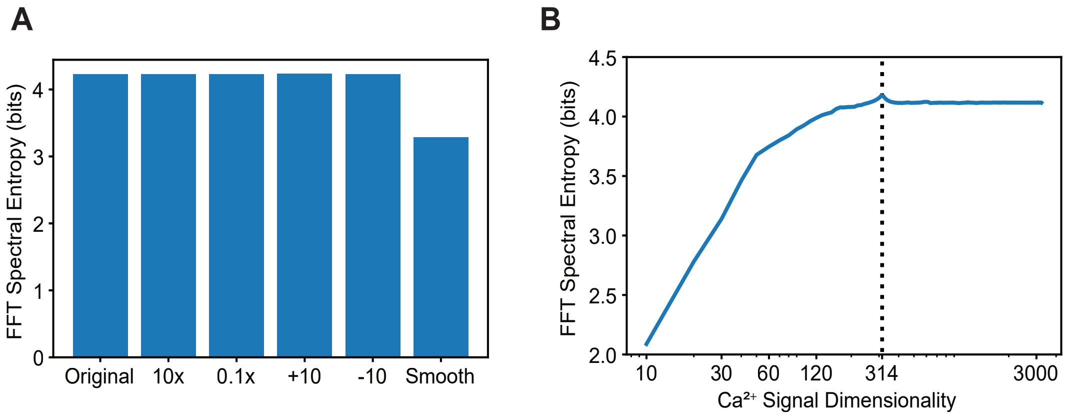

Appendix Figure S4: FFT Spectral Entropy Robustness. A) FFT spectral entropy is robust to translations and rescaling; unlike other measures of differential entropy, it does not depend on the scale of the data. Smoothing the data by convolution expectedly results in a loss of information due to removal of high frequencies. B) The dotted black line shows the true dimensionality and the blue line shows spectral entropy as a function of dimensionality. FFT spectral entropy is robust to interpolations that change the dimensionality of the data that do not affect the distribution of frequencies. Expectedly, reducing dimensionality results in a loss of high frequency information, though most of the information is at relatively low or mid frequencies.

To estimate the amount of extra information assuming no redundancy between elements, we calculated the NRI as a function of gene set size. For $I(G_i;Ca^{2+})$, the NRI is simply defined as $\sum_{i=1}^{83} I(G_i;Ca^{2+})$ because each gene occurs only once. This equation can be generalized to gene sets of any size by dividing $I(\{G_0, \ldots, G_n\};Ca^{2+})$ by the number of times a gene appears in a particular set. Replacing the sum with an expected value multiplied by the number of sets is also useful to avoid having to calculate the value of each element. Equation 2 defines the generalization and is used to calculate the redundancy explained in Equation 3, which represents the fraction of redundant information at a given gene set size.
