# Supplementary material for: Quantifying the phenotypic information in mRNA abundance
Source: Mol Syst Biol. 2022 Aug 15;18(8):e11001. doi: 10.15252/msb.202211001 (PMC9376724; doi:10.15252/msb.202211001)
Supplement: Supplementary file 2 [file MSB-18-e11001-s001.pdf]

Quantifying the phenotypic information in mRNA abundance

Evan Maltz and Roy Wollman
DOI: 10.15252/msb.202211001

Corresponding author(s): Roy Wollman (rwollman@ucla.edu)

Review Timeline:

Submission Date:	24th Feb 22
Editorial Decision:	29th Mar 22
Revision Received:	18th May 22
Editorial Decision:	11th Jul 22
Revision Received:	13th Jul 22
Accepted:	14th Jul 22

Editor: Jingyi Hou

Transaction Report:

(Note: With the exception of the correction of typographical or spelling errors that could be a source of ambiguity, letters and reports are not edited. Depending on transfer agreements, referee reports obtained elsewhere may or may not be included in this compilation. Referee reports are anonymous unless the Referee chooses to sign their reports.)

1st Editorial Decision 29th Mar 2022

Thank you for submitting your work to Molecular Systems Biology. We have now heard back from the three reviewers who agreed to evaluate your study. As you will see below, the reviewers find the topic of your study interesting. They raise however a series of concerns, which we would ask you to address in a major revision.

I think the reviewers' recommendations are relatively straightforward, so there is no need to reiterate all the points listed below. In light of the comments from Reviewers #2 and #3, we would ask you to edit the manuscript to make sure that the main findings and methodology are sufficiently clear and easily accessible to the general audience of Molecular Systems Biology.

All other issues raised by the reviewers need to be satisfactorily addressed. As you may already know, our editorial policy allows in principle a single round of major revision. It is therefore essential to provide responses to the reviewers' comments that are as complete as possible.

On a more editorial level, we would ask you to address the following issues:

REFEREE REPORTS

Reviewer #1:

Review: Quantifying the phenotypic information in mRNA abundance

In this manuscript, Wollman and colleagues carry out an important analysis, namely, to quantify the mutual information between transcript abundances and phenotypic behaviour in single cells. Such studies are in fact long overdue, given the huge popularity in single-cell RNA sequencing techniques and the often-implicit assumption that such datasets can explain all single-cell behaviour. While the authors use only the abundances of 83 genes in single cells, these abundances were determined by MERFISH, which is a lot more sensitive than scRNAseq. Also, these genes have been selected to contain a lot of information about dynamic Ca^{2+} responses. It is therefore all the more striking that they find that transcript abundances only explain 60% of the phenotype, and that 54% of the phenotype (90% of 60%) is explained with 12 genes (!!). This finding doesn't surprise me but is a very important message to the scRNAseq field, as it strongly moderates the expectations from the data generated with this

technology. In addition, the theoretical framework they apply provides a very useful demonstration for how this can be applied to other datasets. I therefore recommend this manuscript for publication in MSB if the authors can address the following comments:

Major Comments:

• When the authors refer to 90% of the captured phenotypic information is captured by a small subset of genes, they imply 90% of 60% of total phenotypic information. It is less confusing if the authors would stick to percentages of total... so 60% and 54%....
• In the abstract the authors refer to 53 genes capturing 54%. Later in the results section the authors show that actually only 12 genes describe the 54%. This can confuse the reader.
• It seems that transcript information only explains 60% of the total spectral entropy. 90% of this information is explained by 12 genes. Critically one could question whether the measurement of the full transcriptome adds information about the phenotype under investigation, as the mutual information gain seems to plateau with 12 genes. It opens up the question whether the information in the transcriptome is generally only partially predictive of the phenotype. This should be pointed out in the discussion. Maybe worth discussing whether additional transcripts would at some point add information. E.g genes that have not been canonically involved in the calcium signalling pathway.

"The magnitude of the correlations for all gene pairs were relatively low with an average of r = 0.16 compared to just cell cycle genes at r = 0.44. One interpretation of the low correlations and heterogeneous transcript distributions is that transcripts contain unique information. To test this hypothesis, we calculated the differential entropy of genes using PCA (Fig 1C-E). Because each principal component is an independent, weighted sum of the row vectors of the data, we can approximate the differential entropy among orthogonal components assuming normality via the central limit theorem. Differential entropy across principal components does not measure the information in absolute terms, but can describe how the information is distributed relative to the explained variance. We found that 6 principal components explain 75% of the variance, but only 15% of the entropy. The contrast between Fig 1C and 1D appears contradictory in that few orthogonal components explain most of the variance, yet entropy is steadily added across components with no obvious plateau. This analysis shows that simple measures such as explained variance that are often used for dimensionality reduction are not necessarily appropriate proxies of information content"

• The approximation of differential entropy using principal components is not clear. Supplementary information on the calculation via central limit theorem would be helpful. Alternatively, the authors should provide reference literature where this method has been applied before.

Figure 2B

• The authors use the sum of mutual information as a proxy for redundant mutual information. It supports the notion of redundancy described in the text but also gives a false impression about the total content of mutual information in the system. Instead, the authors should show whether summing mutual information in this context is applicable mathematically.

Figure 3B

• Figure 3B shows the distribution of binned genes pairs that correspond to redundant, unique and synergistic indices. The authors do not comment on the bin with 0bits SRI index, which should contain unique information, in the text. The authors introduce the connection of SRI=0 to unique information but only comment on the redundant and synergistic part. Would be interesting to know the identity of these genes and their involvement in information about calcium signalling.

• The documented code used in the analysis should be made available.

Minor comments:

• A small remark on consistency. In Figure 1D the axis name is "Differential Entropy" whereas in Figure 4D it is "Gene Differential Entropy"

Reviewer #2:

Maltz & Wollman present a manuscript in which they applied concepts from information theory to interpret MERFISH single-cell gene expression as well as Ca2+ dynamics data collected in cell culture. The authors studied the variance and entropy in differential gene expression, as well as differences in Ca2+ dynamics between cells. Then they moved on to study the mutual information between gene expression and Ca2+ signals, starting with individual genes, moving on to gene pairs, and finally to gene sets. The major findings include that 2.5 (+/- 0.4) bits of calcium signaling information entropy is encoded in gene expression, that gene pairs contain highly redundant information, and that the 12 most informative genes can account for 90 % of the expression / Ca2+ dynamics mutual information.

We think this paper presents an interesting approach and a new perspective for looking at gene expression and phenotypic data,

and therefore is worthy of publication. However, there are some major concerns related to the methodology and interpretation of the data that we believe have to be addressed before this paper can be published.

Concerns related to methodologies, equations, and clarity

Our general comments related to the methodologies are centered on clarity, especially when it comes to how the actual gene expression / Ca2+ dynamics data are utilized and incorporated into the calculations.

---- In the Introduction section, in the second-to-last paragraph, the authors attempt to summarize mutual information neural estimator (MINE) in a few sentences. However, we find the descriptions here very confusing. For example, the authors introduce some new symbols, including G, KL, D_KL, and E, yet provide no definition of these symbols. Could the authors please define all symbols? Along these lines, we find the equations presented here really uninformative and hard to interpret given the author's brief descriptions. Given the central goal of the paper, which is to introduce these concepts to gene-expression-phenotype studies, in which most readers will be unfamiliar with these approaches, we feel it is important the authors provide some simple explanations of these concepts. If the authors intend to summarize MINE here, can the authors attempt to confer the central ideas of MINE with just words? In addition, the authors should consider detailing how they applied MINE to gene expression / Ca2+ dynamics in the Methods section, with more equations and variables that point to the actual data, so that readers understand how the actual expression / signaling data is utilized and incorporated

---- Related to the previous point, we have a general question about how the Ca2+ signaling dynamics is incorporated into the calculation of mutual information. Is the Ca2+ signaling dynamics represented as a vector of Ca2+ magnitudes at various time points, or is it processed into the frequency domain and / or put through other transformations? This can be clarified by showing mathematical equations that incorporate the actual data. Given the central goal of linking gene expression to phenotype, we feel it is important that the methods for quantifying phenotype are very clearly explained.

---- In the discussion related to Fig 1F, the authors claimed that the Ca2+ dynamics periodogram contains 4.2 bits of information. Can the authors show in the Methods section how this number is calculated? One central conclusion to this paper is that the mutual information between gene expression and Ca2+ dynamics accounts (2.5 bits) for 60 % of the information encoded in Ca2+ dynamics (4.2 bits). However, without showing details on how these two numbers are calculated, it is hard to convince readers that it is reasonable to compare these two numbers.

---- The authors introduced the idea of "differential entropy" in the discussion related to Fig. 1. Can the authors define what they mean by differential entropy, and provide mathematical equations that show how it is calculated from the actual gene expression data?

---- In the discussion related to Fig 4C, and in Equations 2 & 3, the authors introduced the concepts of non-redundant information and redundancy explained. In Equation 2, how is the expected value of mutual information of a subset of k genes and Ca2+ dynamics calculated? We also find it hard to understand why this number is then multiplied by the total number of gene subsets of size k from a pool of n, and then divided by the total number of gene subsets of size k-1 from a pool of n-1. Can the authors provide more explanations or justifications for their definition? In Equation 3, is the subscript "k" and "1" missing from the top and bottom G respectively?

Concerns related to data interpretation and clarity

---- In the discussion related to Fig 1B, the authors state that "one interpretation of the low correlations and heterogenous transcript distributions is that transcripts contain unique information." However, we failed to see the logical basis for this argument. Can the authors provide more context or reasonings, or explain what they mean by "unique information"? For example, would one not expect that noise in gene expression lead to both a lack of information per gene and a lack of correlation? In which case, the conclusion that the genes carry unique information would, perhaps, be too strong.

---- In the discussion related to Fig 2A, the authors say that the "most informative genes" contain 57 % of the mutual information. Can the authors define what is their cutoff for the "most informative genes" vs other genes?

---- In Fig 2D, the authors attempt to show that genes that are more informative of other genes also tend to be informative of Ca2+ signaling dynamics. Can the authors show a Pearson correlation coefficient and p-value for this correlation?

---- In the discussion related to Fig 4A, the authors state that "Pairs within an annotation are more redundant than between annotations, with an average difference of 0.1 bits". We are confused by two points: First, is the SRI color bar the absolute value of average SRIs? We would expect the average SRIs to be mostly negative since the gene pairs are supposed to be redundant. However, the color bar indicates that all the SRIs here are positive. Second, judging from the color of the plot, it is really hard to tell that the diagonal squares are more blue than the off-diagonal squares. This overall trend (difference of 0.1 bit) could also be biased by the SRI within the Ca2+ / ER group alone, since it is a lot more blue than the SRI between Ca2+ / ER and other

groups. Can the authors show whether the difference between diagonal and off-diagonal squares here is actually statistically significant?

---- In addition, the authors make the case that the fast time dynamics of Ca2+ signaling make it an ideal phenotype to link to mRNA expression. The rationale, as we understand it, is that mRNA dynamics (transcription and degradation) are often faster than the dynamics of proteins and the phenotypes they cause. This time-scale mismatch would naturally degrade the mutual information between the instantons number of mRNAs from any genes and any given phenotype. However, is this argument not still true for Ca2+ dynamics, as the fast Ca2+ signaling properties are set by the overall abundance of each of the protein components that control aspects of Ca2+ dynamics? Perhaps the authors could clarify this point.

---- Finally, one of the key deliverables of this manuscript is the quantitative value of the number of bits of information conveyed by individual genes (or pairs of genes) for the Ca2+ dynamics within cells. Again, we find the goal of providing a quantitative measure of the information between gene expression and phenotype to be quite compelling; however, without some context, these values are difficult to interpret. Could they authors provide some deeper meaning to the specific number of bits of information they report? At the very least could they comment on whether this amount of information would be considered to be modest or substantial? Or perhaps they could comment on the link between the amount of mutual information and the ability to predict, at a given level of fidelity, the phenotype of a cell given its gene expression.

In addition, we have the following minor concerns:

---- The authors introduced "FFT spectral entropy" in the discussion related to Fig 1F. Can the authors define the acronym FFT as it first appears in the text?

---- Fig 1B title: "a histogram of the pairwise gene correlation matrix (tri-up) ..." We don't believe the shape of the matrix, tri-up, is necessary to be mentioned here. In addition, the shorthand "tri-up" is confusing to readers

---- Is the blue line in Fig 4D a replication of Fig 1D? If so, the authors should state so just in case it confuses the readers

Reviewer #3:

Maltz and Wollman perform a computational study in which they analyse a previously published dataset of paired, single-cell transcriptomic and signalling dynamics in MCF10A cells. They apply a previously published method, MINE, to quantify the causal relationship between mRNA levels and single-cell calcium signalling dynamics. The novelty of this work lies in the application of the analytic framework to this kind of problems. While the analysis tackles a long-standing issue of relating mRNA abundance to dynamic phenotypes at the single-cell level, I feel the results are rather thin and that in the current form the analysis is too technical and more suitable for a specialised journal.

My major concern is the exposition, which I found rushed and difficult to follow for a broader audience of the MSB journal. The introduction takes 3 full pages and includes a very dense description of the methodology. There is no intuitive introduction of terms like "signal entropy" or "differential entropy". The results section starts with the calculation of pairwise correlations for all gene pairs. However, there's very little explanation as for why and what is actually correlated. As is often the case with information theory applied to biological problems the authors do not discuss or explain what the quantification with bits actually mean. Is, e.g., 4.2 bits a lot?

Also, I'd welcome a better explanation of the relationship between bits and the percentage of the total MI. E.g., authors write:

> Most individual genes contain significant information about Ca2+ signals, an average of 0.7 bits,
> and the most informative genes individually account for 57% of the total mutual information between
> all genes and Ca2+ which is 2.5 +/- 0.4 bits.

And another example:

> On average, gene pairs share 0.43 bits which accounts for 61% of the phenotypic information
> contained in the average individual gene.

How do they arrive at 57% or 61%?

The supplementary material is recalled only once and only in the discussion, even though I feel it would be quite beneficial to discuss it before the results. Especially the analysis performed on synthetic data shown in supplementary figure 1 could provide the reader more intuition about the method. Another issue is that the authors casually throw jargon like "information synergy and redundancy" (Discussion) but again, it doesn't mean anything to this reader.

Overall, the paper reads like a technical report and there's insufficient "guidance" of the reader through all the concepts, which makes it difficult to gather the significance of the result.

We thank the reviewers for their positive comments and constructive suggestions. Below we address the reviewers' points inline. In black, we show the original text with changes highlighted in red (track changes). Blue color shows reviewer comments.

Reviewer 1

• When the authors refer to 90% of the captured phenotypic information is captured by a small subset of genes, they imply 90% of 60% of total phenotypic information. It is less confusing if the authors would stick to percentages of total... so 60% and 54%....

We thank the reviewer for this clarifying suggestion and have adjusted the percentages accordingly:

Using different heuristics, we estimated the dependency between the size of a gene set and its information content, revealing that on average a set of 53 genes contains 54% of the information about Ca^{2+} signaling.

We found that 6 principal components explain 75% of the variance, but only 15% of the gene entropy

Most individual genes contain significant information about Ca^{2+} signals, an average of 0.7 bits, and the most informative gene accounts for 34% of the 4.2 bits of signal entropy.

The average mutual information between a single gene and Ca2+ signals is 0.7 bits, which is 17% of the signal entropy (Fig 2C). How the mutual information is shared across genes is not immediately clear.

Each set size was sampled 4 times. For random sets, 53 genes contained 54% of the phenotypic information

The upper bound in green shows that the information quickly plateaus as the best 12 genes contain 54% of the phenotypic information, and all further additions contribute minimal additional information.

• In the abstract the authors refer to 53 genes capturing 54%. Later in the results section the authors show that actually only 12 genes describe the 54%. This can confuse the reader.

We thank the reviewer for this useful revision and have clarified the relationship between the average set of 53 genes and the best set of 12 genes.

Compared to random gene sets, using only the most informative combination of genes dramatically reduces the number of genes required to recapitulate most of the phenotypic information from 53 to 12.

In agreement with Fig 4C, phenotypic information saturates quickly with only 3 principal components accounting for 74% of the 2.5 bits of mutual information between transcripts and signals.

In the best case, only 12 genes contain 54% of signal information, which is significantly fewer than an equally informative 53 random genes.

• It seems that transcript information only explains 60% of the total spectral entropy. 90% of this information is explained by 12 genes. Critically one could question whether the measurement of the full transcriptome adds information about the phenotype under investigation, as the mutual information gain seems to plateau with 12 genes. It opens up the question whether the information in the transcriptome is generally only partially predictive of the phenotype. This should be pointed out in the discussion. Maybe worth discussing whether additional transcripts would at some point add information. E.g genes that have not been canonically involved in the calcium signalling pathway.

We thank the reviewer for noting this important point and have added our thoughts about the contribution of additional gene measurements in the discussion.

Additionally, it is possible that the full transcriptome may contain more phenotypic information than is found in just the 83 genes measured in this study. While only 12 genes accounted for most of the shared information, the apparent plateau and informational redundancy may result from the strong functional relationships and dependencies in the selected gene set. Including significantly more genes related to other cellular processes may provide more information about the observed phenotype by better defining the transcriptional state or revealing indirect dependencies to other cellular processes.

• The approximation of differential entropy using principal components is not clear. Supplementary information on the calculation via central limit theorem would be helpful. Alternatively, the authors should provide reference literature where this method has been applied before.

We thank the reviewer for this helpful suggestion and have added the calculation in the methods section:

Differential Entropy: Differential entropy was calculated via the determinant of the covariance matrix of the PCA-transformed data. This approach was used to estimate the entropy of the mRNA transcript counts.

$$\frac{n}{2} + \frac{n log_2 2\pi}{2} + det\Sigma_{PC} \qquad (2)$$

n = number of principal components, Σ = the covariance matrix, *PC* = PCA-transformed data for a given *n*

Figure 2B

• The authors use the sum of mutual information as a proxy for redundant mutual information. It supports the notion of redundancy described in the text but also gives a false impression about

the total content of mutual information in the system. Instead, the authors should show whether summing mutual information in this context is applicable mathematically.

We thank the reviewer for noting the need to contextualize the sum of mutual information and have clarified its use in estimating redundant information.

If each gene contained completely unique information, then the sum of the phenotypic information in each gene should add up to the total of 2.5 +/- 0.4 bits. Interestingly, this sum is significantly larger than the total $I(G;Ca^{2+})$, indicating a high degree of redundancy (Fig 2B).

Figure 3B

• Figure 3B shows the distribution of binned genes pairs that correspond to redundant, unique and synergistic indices. The authors do not comment on the bin with 0bits SRI index, which should contain unique information, in the text. The authors introduce the connection of SRI=0 to unique information but only comment on the redundant and synergistic part. Would be interesting to know the identity of these genes and their involvement in information about calcium signalling.

We thank the reviewer for this suggestion and have added a reference to the genes we find with SRI=0.

Most of the genes with near zero SRI were generally uninformative about signaling based on Fig 2A.

• The documented code used in the analysis should be made available.

Minor comments:

• A small remark on consistency. In Figure 1D the axis name is "Differential Entropy" whereas in Figure 4D it is "Gene Differential Entropy"

We thank the reviewer for this suggestion to increase consistency. Figure 4D has been changed to “Differential Entropy” for consistency.

Reviewer 2

Concerns related to methodologies, equations, and clarity

Our general comments related to the methodologies are centered on clarity, especially when it comes to how the actual gene expression / Ca2+ dynamics data are utilized and incorporated into the calculations.

---- In the Introduction section, in the second-to-last paragraph, the authors attempt to summarize mutual information neural estimator (MINE) in a few sentences. However, we find the descriptions here very confusing. For example, the authors introduce some new symbols, including G, KL, D_KL, and E, yet provide no definition of these symbols. Could the authors please define all symbols? Along these lines, we find the equations presented here really uninformative and hard to interpret given the author's brief descriptions. Given the central goal

of the paper, which is to introduce these concepts to gene-expression-phenotype studies, in which most readers will be unfamiliar with these approaches, we feel it is important the authors provide some simple explanations of these concepts. If the authors intend to summarize MINE here, can the authors attempt to confer the central ideas of MINE with just words? In addition, the authors should consider detailing how they applied MINE to gene expression / Ca2+ dynamics in the Methods section, with more equations and variables that point to the actual data, so that readers understand how the actual expression / signaling data is utilized and incorporated

We thank the reviewer for this clarifying suggestion and have added all relevant definitions and expanded our descriptions of MINE.

Briefly, MINE is a universal function approximator that searches for a mapping function $\boldsymbol{T}$ in a large space of encoder functions parameterized by $\boldsymbol{\theta}$. $\boldsymbol{T}$ maps the data consisting of mRNA counts and Ca^{2+} signal dynamics, $\boldsymbol{G}$ and $\boldsymbol{Ca^{2+}}$ respectively, of arbitrary dimensionality such that $\boldsymbol{T_\theta : (G, Ca^{2+}) \longrightarrow \mathbb{R}}$. Remarkably, the data require no preprocessing, MINE is simply trained on the raw data because all transformations required for an efficient mapping are theoretically learnable by a model with enough parameters and samples. Letting $\mathbb{P} := \mathbb{P}_{G,Ca^{2+}}$ represent the joint probability, i.e. paired data, and $\mathbb{Q} := \mathbb{P}_G \otimes \mathbb{P}_{Ca^{2+}}$ represent the product of the marginal probabilities, i.e. independently sampled data, the mutual information between $\boldsymbol{G}$ and $\boldsymbol{Ca^{2+}}$ is the distance between the joint and marginal distributions. This distance is measured using the Kullback-Leibler divergence (D_{KL}), and a stronger relationship between $\boldsymbol{G}$ and $\boldsymbol{Ca^{2+}}$ is equivalent to a greater distance between the joint and marginals: $\boldsymbol{I(G, Ca^{2+}) = D_{KL}(\mathbb{P}||\mathbb{Q})}$. Using the Donsker-Varadhan representation of the D_{KL}, the model parameters $\boldsymbol{\theta}$ are optimal when gradient ascent has maximized $\boldsymbol{\mathbb{E}_{\mathbb{P}}[T_\theta] - log(\mathbb{E}_{\mathbb{Q}}[e^{T_\theta}]) \leq D_{KL}(\mathbb{P}||\mathbb{Q})}$, where $\mathbb{E}$ denotes the expected value.This estimate represents a lower bound on the mutual information.

---- Related to the previous point, we have a general question about how the Ca2+ signaling dynamics is incorporated into the calculation of mutual information. Is the Ca2+ signaling dynamics represented as a vector of Ca2+ magnitudes at various time points, or is it processed into the frequency domain and / or put through other transformations? This can be clarified by showing mathematical equations that incorporate the actual data. Given the central goal of linking gene expression to phenotype, we feel it is important that the methods for quantifying phenotype are very clearly explained.

We thank the reviewer for this suggestion and have clarified how the data is used by the model for the mutual information estimation (see above).

---- In the discussion related to Fig 1F, the authors claimed that the Ca2+ dynamics periodogram contains 4.2 bits of information. Can the authors show in the Methods section how this number is calculated? One central conclusion to this paper is that the mutual information between gene expression and Ca2+ dynamics accounts (2.5 bits) for 60 % of the information encoded in Ca2+ dynamics (4.2 bits). However, without showing details on how these two

numbers are calculated, it is hard to convince readers that it is reasonable to compare these two numbers.

We thank the reviewer for this suggestion and have included a description of the spectral entropy calculation in the methods.

Spectral Entropy. Spectral entropy of a signal is defined as the Shannon entropy (*H*) of the normalized power spectral density (*P*). Although the calculation requires a sampling frequency (f_s), the result does not change above a sufficiently large value.

$$H(f_s) = -\sum_{f=1}^{f_s/2} P(f) log_2 P(f) \tag{1}$$

---- The authors introduced the idea of "differential entropy" in the discussion related to Fig. 1. Can the authors define what they mean by differential entropy, and provide mathematical equations that show how it is calculated from the actual gene expression data?

We thank the reviewer for this suggestion and have included a description of the differential entropy calculation in the methods.

Differential Entropy: Differential entropy was calculated via the determinant of the covariance matrix of the PCA-transformed data. This approach was used to estimate the entropy of the mRNA transcript counts.

$$\frac{n}{2} + \frac{n log_2 2\pi}{2} + det\Sigma_{PC} \tag{2}$$

n = number of principal components, Σ = the covariance matrix, *PC* = PCA-transformed data for a given *n*

---- In the discussion related to Fig 4C, and in Equations 2 & 3, the authors introduced the concepts of non-redundant information and redundancy explained. In Equation 2, how is the expected value of mutual information of a subset of k genes and Ca2+ dynamics calculated? We also find it hard to understand why this number is then multiplied by the total number of gene subsets of size k from a pool of n, and then divided by the total number of gene subsets of size k-1 from a pool of n-1. Can the authors provide more explanations or justifications for their definition? In Equation 3, is the subscript "k" and "1" missing from the top and bottom G respectively?

We thank the reviewer for noting the lack of clarity in our description of the redundancy explained calculations. We have added more context and justification for this calculation.

Redundancy Explained. This metric represents the amount of extra information assuming no redundancy between elements. Equation 4 first calculates an expected value by taking the mean of sampled gene sets of size k. The expected value is multiplied by the number of possible sets then divided the number of times an individual gene appears in all sets to calculate the

$$NRI = \frac{\mathbb{E}[I(\{G_1, \ldots, G_k\}; Ca^{2+})]\binom{n}{k}}{\binom{n-1}{k-1}} = \mathbb{E}[I(\{G_1, \ldots, G_k\}; Ca^{2+})]\frac{n}{k}$$

nonredundant information (NRI) as if all individual sets contain unique information:

(4)

k = set size, n = total number of genes

From Equation 4, we can calculate the fraction of purely redundant information for a set of size k out of the maximum, which is the NRI at k=1 minus the full mutual information. The Redundancy Explained (RE) is calculated as follows:

$$RE = 1 - \frac{NRI_k - I(G;Ca^{2+})}{NRI_1 - I(G;Ca^{2+})} \tag{5}$$

Concerns related to data interpretation and clarity

---- In the discussion related to Fig 1B, the authors state that "one interpretation of the low correlations and heterogenous transcript distributions is that transcripts contain unique information." However, we failed to see the logical basis for this argument. Can the authors provide more context or reasonings, or explain what they mean by "unique information"? For example, would one not expect that noise in gene expression lead to both a lack of information per gene and a lack of correlation? In which case, the conclusion that the genes carry unique information would, perhaps, be too strong.

We thank the reviewer for this request to provide context and have clarified that our interpretation depends on most genes being generally informative, which is justified in Figure 2A.

Assuming most genes are generally informative, one interpretation of the low correlations and heterogeneous transcript distributions is that transcripts contain unique information.

---- In the discussion related to Fig 2A, the authors say that the "most informative genes" contain 57 % of the mutual information. Can the authors define what is their cutoff for the "most informative genes" vs other genes?

We thank the reviewer for noting this lack of clarity and have changed the text to indicate that we are referring to the most informative gene in Figure 2A.

and the most informative gene accounts for 34%

---- In the discussion related to Fig 4A, the authors state that "Pairs within an annotation are more redundant than between annotations, with an average difference of 0.1 bits". We are

confused by two points: First, is the SRI color bar the absolute value of average SRIs? We would expect the average SRIs to be mostly negative since the gene pairs are supposed to be redundant. However, the color bar indicates that all the SRIs here are positive. Second, judging from the color of the plot, it is really hard to tell that the diagonal squares are more blue than the off-diagonal squares. This overall trend (difference of 0.1 bit) could also be biased by the SRI within the Ca2+ / ER group alone, since it is a lot more blue than the SRI between Ca2+ / ER and other groups. Can the authors show whether the difference between diagonal and off-diagonal squares here is actually statistically significant?

We thank the reviewer for this clarifying suggestion. We have added some space between the tick marks and the values to more clearly indicate that the values are negative on the color bar. We have also removed the statement about the difference between diagonal and off-diagonal as the results were not statistically significant.

---- In addition, the authors make the case that the fast time dynamics of Ca2+ signaling make it an ideal phenotype to link to mRNA expression. The rationale, as we understand it, is that mRNA dynamics (transcription and degradation) are often faster than the dynamics of proteins and the phenotypes they cause. This time-scale mismatch would naturally degrade the mutual information between the instantons number of mRNAs from any genes and any given phenotype. However, is this argument not still true for Ca2+ dynamics, as the fast Ca2+ signaling properties are set by the overall abundance of each of the protein components that control aspects of Ca2+ dynamics? Perhaps the authors could clarify this point.

We thank the reviewer for these questions and have clarified our rationale regarding timescale separation.

Ca^{2+} signaling is a system in which the emerging phenotype is faster than changes in mRNA abundance. This timescale separation allows us to assume mRNA abundances are at a quasi-steady state and do not change significantly during the experiment.

---- Finally, one of the key deliverables of this manuscript is the quantitative value of the number of bits of information conveyed by individual genes (or pairs of genes) for the Ca2+ dynamics within cells. Again, we find the goal of providing a quantitative measure of the information between gene expression and phenotype to be quite compelling; however, without some context, these values are difficult to interpret. Could they authors provide some deeper meaning to the specific number of bits of information they report? At the very least could they comment on whether this amount of information would be considered to be modest or substantial? Or perhaps they could comment on the link between the amount of mutual information and the ability to predict, at a given level of fidelity, the phenotype of a cell given its gene expression.

We thank the reviewer for this suggestion and have provided a discussion of the intuition for the importance of the number of bits we report.

Even though correlations between mRNA and protein levels are generally low, a substantial amount of phenotypically relevant information is still preserved in the transcriptome. Our results support the use of mRNA measurements to infer and predict useful phenotypic characteristics of

cell populations. One interpretation of the 2.5 (+/- 0.4) bits of mutual information is that transcripts can differentiate approximately 6 distinct states of Ca^{2+} signaling dynamics.

In addition, we have the following minor concerns:

---- The authors introduced "FFT spectral entropy" in the discussion related to Fig 1F. Can the authors define the acronym FFT as it first appears in the text?

We thank the reviewer for this suggestion and have removed unused initialisms and better defined spectral entropy in the methods.

Differential entropy of Ca^{2+} can be estimated using spectral entropy (Equation 1), a scale-invariant measure of information (Burg, 1975).

Spectral entropy of a signal is defined as the Shannon entropy (H) of the normalized power spectral density (P), calculated here using the Fourier transform.

---- Fig 1B title: "a histogram of the pairwise gene correlation matrix (tri-up) ..." We don't believe the shape of the matrix, tri-up, is necessary to be mentioned here. In addition, the shorthand "tri-up" is confusing to readers

We thank the reviewer for this suggestion and have removed the confusing terminology.

B) A histogram of the pairwise gene correlation matrix which highlights the relatively low correlations.

---- Is the blue line in Fig 4D a replication of Fig 1D? If so, the authors should state so just in case it confuses the readers

We thank the reviewer for this suggestion and have included a reference to Figure 1D in the main text.

Finally, we calculated the mutual information between transcript principal components and Ca^{2+} signals to compare with differential entropy (from Fig 1D) and understand how useful phenotypic information is distributed (Fig 4D).

Reviewer 3

There is no intuitive introduction of terms like "signal entropy" or "differential entropy". The results section starts with the calculation of pairwise correlations for all gene pairs. However, there's very little explanation as for why and what is actually correlated. As is often the case with information theory applied to biological problems the authors do not discuss or explain what the quantification with bits actually mean. Is, e.g., 4.2 bits a lot?

We thank the reviewer for this suggestion and we have included better definitions of signal entropy and differential entropy as well as provided context about the 4.2 bits.

Signal entropy can be calculated using this distribution of frequencies, which we found to be 4.2 bits or ~18 signaling states.

To test this hypothesis, we quantified the transcriptional information by performing PCA then calculating the differential entropy, a measure of information for continuous probability distributions, of the components (Equation 2, Fig 1C-E).

To estimate the signal entropy, i.e. information content in Ca^{2+} signaling, we took advantage of its dynamic patterns.

Also, I'd welcome a better explanation of the relationship between bits and the percentage of the total MI. E.g., authors write:

> Most individual genes contain significant information about Ca2+ signals, an average of 0.7 bits,
> and the most informative genes individually account for 57% of the total mutual information between
> all genes and Ca2+ which is 2.5 +/- 0.4 bits.

And another example:

> On average, gene pairs share 0.43 bits which accounts for 61% of the phenotypic information
> contained in the average individual gene.

How do they arrive at 57% or 61%?

We thank the reviewer for this suggestion and have clarified the relationship between the percentages and bits.

Most individual genes contain significant information about Ca^{2+} signals, an average of 0.7 bits, and the most informative gene accounts for 34% of the 4.2 bits of signal entropy.

On average, gene pairs share 0.43 bits which accounts for 61% of the 0.7 bits of phenotypic information contained in the average individual gene.

The supplementary material is recalled only once and only in the discussion, even though I feel it would be quite beneficial to discuss it before the results. Especially the analysis performed on synthetic data shown in supplementary figure 1 could provide the reader more intuition about the method. Another issue is that the authors casually throw jargon like "information synergy and redundancy" (Discussion) but again, it doesn't mean anything to this reader.

We thank the reviewer for this suggestion and have included a reference to the appendix in the main text.

To help choose hyperparameters and evaluate MINE's performance, we tested the model on multivariate gaussian distributions and found a mean residual of 0.37 bits with a Pearson correlation coefficient of 0.97 to the ground truth (Appendix Figure S2).

Thank you for sending us your revised manuscript. We have now heard back from the three reviewers who agreed to evaluate your study. As you will see, the reviewers are satisfied with the modifications made and think the study is now suitable for publication.

Before we can formally accept your manuscript, we would ask you to address the following editorial-level issues:

REFEREE REPORTS

Reviewer #1:

The authors have addressed all our comments and I recommend the manuscript for publication in MSB.

Reviewer #2:

We thank the authors for going through our point-by-point concerns, and addressing each one of them. While we still have some lingering concerns regarding the clarity and sufficiency of some of these responses, we believe the paper is now suitable for publication.

Reviewer #3:

All my concerns have been sufficiently addressed by the authors. The manuscript's clarity has improved.

The authors have made all requested editorial changes.

,

Thank you again for sending us your revised manuscript. We are now satisfied with the modifications made and I am pleased to inform you that your paper has been accepted for publication.

EMBO Press Author Checklist

Corresponding Author Name: Roy Wollman	
Journal Submitted to: Molecular Systems Biology	
Manuscript Number: MSB-2022-11001R	

USEFUL LINKS FOR COMPLETING THIS FORM
The EMBO Journal - Author Guidelines
EMBO Reports - Author Guidelines
Molecular Systems Biology - Author Guidelines
EMBO Molecular Medicine - Author Guidelines

Reporting Checklist for Life Science Articles (updated January 2022)
This checklist is adapted from Materials Design Analysis Reporting (MDAR) Checklist for Authors. MDAR establishes a minimum set of requirements in transparent reporting in the life sciences (see Statement of Task: 10.31222/osf.io/9sm4x). Please follow the journal's guidelines in preparing your manuscript.
Please note that a copy of this checklist will be published alongside your article.

Abridged guidelines for figures
1. Data
The data shown in figures should satisfy the following conditions:
- the data were obtained and processed according to the field's best practice and are presented to reflect the results of the experiments in an accurate and unbiased manner.
- ideally, figure panels should include only measurements that are directly comparable to each other and obtained with the same assay.
- plots include clearly labeled error bars for independent experiments and sample sizes. Unless justified, error bars should not be shown for technical replicates.
- if n<5, the individual data points from each experiment should be plotted. Any statistical test employed should be justified.
- Source Data should be included to report the data underlying figures according to the guidelines set out in the authorship guidelines on Data Presentation.

2. Captions
Each figure caption should contain the following information, for each panel where they are relevant:
- a specification of the experimental system investigated (eg cell line, species name).
- the assay(s) and method(s) used to carry out the reported observations and measurements.
- an explicit mention of the biological and chemical entity(ies) that are being measured.
- an explicit mention of the biological and chemical entity(ies) that are altered/varied/perturbed in a controlled manner.
- the exact sample size (n) for each experimental group/condition, given as a number, not a range;
- a description of the sample collection allowing the reader to understand whether the samples represent technical or biological replicates (including how many animals, litters, cultures, etc.).
- a statement of how many times the experiment shown was independently replicated in the laboratory.
- definitions of statistical methods and measures:
  - common tests, such as t-test (please specify whether paired vs. unpaired), simple χ^2 tests, Wilcoxon and Mann-Whitney tests, can be unambiguously identified by name only, but more complex techniques should be described in the methods section;
  - are tests one-sided or two-sided?
  - are there adjustments for multiple comparisons?
  - exact statistical test results, e.g., P values = x but not P values < x;
  - definition of 'center values' as median or average;
  - definition of error bars as s.d. or s.e.m.

Please complete ALL of the questions below.
Select "Not Applicable" only when the requested information is not relevant for your study.

Materials

Newly Created Materials	Information included in the manuscript?	In which section is the information available? (Reagents and Tools Table, Materials and Methods, Figures, Data Availability Section)
New materials and reagents need to be available; do any restrictions apply?	Not Applicable	

Antibodies	Information included in the manuscript?	In which section is the information available? (Reagents and Tools Table, Materials and Methods, Figures, Data Availability Section)
For **antibodies** provide the following information:<br>- Commercial antibodies: RRID (if possible) or supplier name, catalogue number and or/clone number<br>- Non-commercial: RRID or citation	Not Applicable	

DNA and RNA sequences	Information included in the manuscript?	In which section is the information available? (Reagents and Tools Table, Materials and Methods, Figures, Data Availability Section)
**Short novel DNA or RNA including primers, probes**: provide the sequences.	Not Applicable	

Cell materials	Information included in the manuscript?	In which section is the information available? (Reagents and Tools Table, Materials and Methods, Figures, Data Availability Section)
**Cell lines:** Provide species information, strain. Provide accession number in repository **OR** supplier name, catalog number, clone number, and/**OR** RRID.	Not Applicable	
**Primary cultures:** Provide species, strain, sex of origin, genetic modification status.	Not Applicable	
Report if the cell lines were recently **authenticated** (e.g., by STR profiling) and tested for mycoplasma contamination.	Not Applicable	

Experimental animals	Information included in the manuscript?	In which section is the information available? (Reagents and Tools Table, Materials and Methods, Figures, Data Availability Section)
**Laboratory animals or Model organisms:** Provide species, strain, sex, age, genetic modification status. Provide accession number in repository **OR** supplier name, catalog number, clone number, **OR** RRID.	Not Applicable	
**Animal observed in or captured from the field:** Provide species, sex, and age where possible.	Not Applicable	
Please detail **housing and husbandry conditions**.	Not Applicable	

Plants and microbes	Information included in the manuscript?	In which section is the information available? (Reagents and Tools Table, Materials and Methods, Figures, Data Availability Section)
**Plants:** provide species and strain, ecotype and cultivar where relevant, unique accession number if available, and source (including location for collected wild specimens).	Not Applicable	
**Microbes:** provide species and strain, unique accession number if available, and source.	Not Applicable	

Human research participants	Information included in the manuscript?	In which section is the information available? (Reagents and Tools Table, Materials and Methods, Figures, Data Availability Section)
If collected and within the bounds of privacy constraints report on age, sex and gender or ethnicity for all study participants.	Not Applicable	

Core facilities	Information included in the manuscript?	In which section is the information available? (Reagents and Tools Table, Materials and Methods, Figures, Data Availability Section)
If your work benefited from core facilities, was their service mentioned in the acknowledgments section?	Not Applicable	

Design

Study protocol	Information included in the manuscript?	In which section is the information available? (Reagents and Tools Table, Materials and Methods, Figures, Data Availability Section)
If study protocol has been **pre-registered, provide DOI in the manuscript**. For clinical trials, provide the trial registration number **OR** cite DOI.	Not Applicable	
Report the **clinical trial registration number** (at ClinicalTrials.gov or equivalent), where applicable.	Not Applicable	

Laboratory protocol	Information included in the manuscript?	In which section is the information available? (Reagents and Tools Table, Materials and Methods, Figures, Data Availability Section)
Provide DOI OR other citation details if **external detailed step-by-step protocols** are available.	Not Applicable	

Experimental study design and statistics	Information included in the manuscript?	In which section is the information available? (Reagents and Tools Table, Materials and Methods, Figures, Data Availability Section)
Include a statement about **sample size** estimate even if no statistical methods were used.	Not Applicable	
Were any steps taken to minimize the effects of subjective bias when allocating animals/samples to treatment (e.g. **randomization procedure**)? If yes, have they been described?	Not Applicable	
Include a statement about **blinding** even if no blinding was done.	Not Applicable	
Describe **inclusion/exclusion criteria** if samples or animals were excluded from the analysis. Were the criteria pre-established?<br><br>If sample or data points were omitted from analysis, report if this was due to attrition or intentional exclusion and provide justification.	Not Applicable	
For every figure, are **statistical tests** justified as appropriate? Do the data meet the assumptions of the tests (e.g., normal distribution)? Describe any methods used to assess it. Is there an estimate of variation within each group of data? Is the variance similar between the groups that are being statistically compared?	Yes	Material and methods

Sample definition and in-laboratory replication	Information included in the manuscript?	In which section is the information available? (Reagents and Tools Table, Materials and Methods, Figures, Data Availability Section)
In the figure legends: state number of times the experiment was **replicated** in laboratory.	Not Applicable	
In the figure legends: define whether data describe **technical or biological replicates**.	Not Applicable	

Ethics

Ethics	Information included in the manuscript?	In which section is the information available? (Reagents and Tools Table, Materials and Methods, Figures, Data Availability Section)
Studies involving **human participants**: State details of **authority granting ethics approval** (IRB or equivalent committee(s), provide reference number for approval.	Not Applicable	
Studies involving **human participants**: Include a statement confirming that **informed consent** was obtained from all subjects and that the experiments conformed to the principles set out in the WMA Declaration of Helsinki and the Department of Health and Human Services Belmont Report.	Not Applicable	
Studies involving **human participants:** For publication of **patient photos**, include a statement confirming that consent to publish was obtained.	Not Applicable	
Studies involving experimental **animals**: State details of **authority granting ethics approva**l (IRB or equivalent committee(s), provide reference number for approval. Include a statement of compliance with ethical regulations.	Not Applicable	
Studies involving **specimen and field samples:** State if relevant **permits** obtained, provide details of authority approving study; if none were required, explain why.	Not Applicable	

Dual Use Research of Concern (DURC)	Information included in the manuscript?	In which section is the information available? (Reagents and Tools Table, Materials and Methods, Figures, Data Availability Section)
Could your study fall under dual use research restrictions? Please check biosecurity documents and list of **select agents and toxins** (CDC): https://www.selectagents.gov/sat/list.htm	Not Applicable	
If you used a select agent, is the security level of the lab appropriate and reported in the manuscript?	Not Applicable	
If a study is subject to dual use research of concern regulations, is the name of the **authority granting approval and reference number** for the regulatory approval provided in the manuscript?	Not Applicable	

Reporting

The MDAR framework recommends adoption of discipline-specific guidelines, established and endorsed through community initiatives. Journals have their own policy about requiring specific guidelines and recommendations to complement MDAR.

Adherence to community standards	Information included in the manuscript?	In which section is the information available? (Reagents and Tools Table, Materials and Methods, Figures, Data Availability Section)
State if relevant guidelines or checklists (e.g., **ICMJE, MIBBI, ARRIVE, PRISMA**) have been followed or provided.	Not Applicable	
For **tumor marker prognostic studies,** we recommend that you follow the **REMARK** reporting guidelines (see link list at top right). See author guidelines, under 'Reporting Guidelines'. Please confirm you have followed these guidelines.	Not Applicable	
For **phase II and III randomized controlled trials**, please refer to the **CONSORT** flow diagram (see link list at top right) and submit the CONSORT checklist (see link list at top right) with your submission. See author guidelines, under 'Reporting Guidelines'. Please confirm you have submitted this list.	Not Applicable	

Data Availability

Data availability	Information included in the manuscript?	In which section is the information available? (Reagents and Tools Table, Materials and Methods, Figures, Data Availability Section)
Have **primary datasets** been deposited according to the journal's guidelines (see 'Data Deposition' section) and the respective accession numbers provided in the Data Availability Section?	Not Applicable	
Were **human clinical and genomic datasets** deposited in a public access-controlled repository in accordance to ethical obligations to the patients and to the applicable consent agreement?	Not Applicable	
Are **computational models** that are central and integral to a study available without restrictions in a machine-readable form? Were the relevant accession numbers or links provided?	Not Applicable	
If publicly available data were reused, provide the respective **data citations in the reference list**.	Not Applicable	
